# Ozone Therapy in Medicine and Dentistry: A Review of the Literature

**DOI:** 10.3390/dj11080187

**Published:** 2023-08-07

**Authors:** Omar A. El Meligy, Noha M. Elemam, Iman M. Talaat

**Affiliations:** 1 Pediatric Dentistry Department, Faculty of Dentistry, King Abdulaziz University, Jeddah 21589, Saudi Arabia; omeligy@kau.edu.sa; 2 Pediatric Dentistry and Dental Public Health Department, Faculty of Dentistry, Alexandria University, Alexandria 21131, Egypt; 3 Clinical Sciences Department, College of Medicine, University of Sharjah, Sharjah 27272, United Arab Emirates; 4 Research Institute of Medical & Health Sciences, University of Sharjah, Sharjah 27272, United Arab Emirates; 5 Pathology Department, Faculty of Medicine, Alexandria University, Alexandria 21131, Egypt

**Keywords:** ozone, clinical applications, medicine, dentistry

## Abstract

Ozone has been successfully used in medicine for over 100 years due to its microbiological qualities. Its powerful oxidation impact, which results in the production of free radicals, and its ability to cause the direct death of nearly all microorganisms is the basis for its bactericide, virucide, and fungicide properties. Ozone also has a medicinal impact that speeds up blood flow and aids wound healing. Ozone may be applied as a gas or dissolved in water for medical purposes. Despite the benefits of using ozone therapeutically, concerns about its use in dentistry still exist. We aimed to provide a summary of the current uses of ozone in medicine and dentistry. An electronic search was performed for all English scientific papers published between 2012 and 2023 using PubMed, Cochrane, and Google Scholar search engines. Ozone, clinical applications, medicine, and dentistry were the search terms used. Seventy full-text articles describing the use of ozone therapy in medicine and dentistry were included in the present review. Ozone has shown several beneficial effects in the medical field. However, despite the encouraging in vitro evidence, the clinical use of ozone in dentistry has not yet been demonstrated as highly effective.

## 1. Introduction

Ozone is a naturally occurring chemical compound consisting of three oxygen atoms. It can absorb most of the ultraviolet energy that the sun emits; thus, it is one of the most significant gases in the stratosphere. As a result, ozone in the stratosphere is essential for both the stratosphere’s thermal structure and the ecological system that supports life on the Earth’s surface [1]. Compared to oxygen, ozone is ten times more soluble in water and 1.6 times denser. It has a half-life of 40 min at 20 °C and is a very unstable compound that breaks into pure oxygen [2].

Ozone is an effective disinfectant with a broad range of activity thanks to its microbiologic and metabolic capabilities. Ozone is an effective and dependable antibacterial agent against bacteria, fungi, protozoa, and viruses, whether in the gaseous or aqueous phase [3,4].

### 1.1. Mode of Action of Ozone

Ozone is known to have numerous effects on the human body. These include bioenergetic, biosynthetic, antibacterial, analgesic, anti-hypoxic, and detoxicating properties [5]. Regarding antibacterial properties, ozone’s oxidant potential causes the breakdown of bacterial and fungal cell walls and cytoplasmic membranes by destroying glycoproteins, glycolipids, and other amino acids. It disrupts the cell’s enzymatic regulatory system and raises membrane permeability, a crucial component of cell viability, causing a quick end to functional activity. Once within the cell, ozone molecules can easily kill bacteria. This effect is general and only affects microbial cells. As a result of their significant antioxidant capacity, they do not harm human body cells. In antibiotic-resistant bacteria, ozone is particularly effective. It exhibits increased antibacterial action in a liquid environment with an acidic pH [6].

Ozone therapy modulates the cellular antioxidant system and the inflammation system. This is due to its ability to produce hydrogen peroxide (H_2_O_2_), aldehydes, and lipid oxidation products (LOPs). Further, this leads to the activation of the nuclear factor erythroid 2-related factor 2 (Nrf2) pathway, which stimulates superoxide dismutase, catalase, glutathione peroxidase, glutathione s–transferase, hem–oxygenase–1, and heat shock protein–70 (HSP70) [7]. Additionally, ozone possesses an anti-inflammatory effect by reducing the activation of the nuclear factor kappa B (NF–κB) pathway, thus decreasing the production of cytokines such as IL–1, IL–2, IL–6, and tumor necrosis factor-alpha (TNF-α) while stimulating the production of cytokines such as IL–4, IL–10, IL–13, and transforming growth factor–beta (TGF–β).

Ozone regulates oxygen metabolism by increasing the red blood cell glycolysis rate, thus increasing the amount of oxygen released to the tissues. Additionally, ozone stimulates the production of enzymes that are free radical scavengers, such as glutathione peroxidase, superoxide dismutase, and catalase [8]. Such an effect was found to be favorable in relieving the oxidative stress observed in diabetes, promoting the use of ozone as an alternative therapy in treating diabetes and its complications [9]. Additionally, ozone was reported to increase airway resistance and respiratory rates and decrease the tidal volume in the lungs [8].

The control of the concentration of ozone administered is a pivotal aspect of ozone therapy, influencing its safety and therapeutic efficacy. Ozone exhibits a range of biological effects that make it valuable in various medical and dental applications. However, maintaining precise and accurate control over the ozone concentration is paramount to avoid potential adverse effects and optimize therapeutic outcomes. Different medical and dental conditions and treatment protocols may require specific ozone concentrations tailored to the patient’s needs. Advanced and reliable ozone delivery systems equipped with precise regulators and monitoring mechanisms are employed to ensure the desired concentration levels are achieved during therapy. Standardized guidelines and best practices are continuously evolving to establish safe and effective ozone therapy protocols, thereby supporting healthcare practitioners in harnessing the full potential of this innovative treatment modality. By adhering to meticulous concentration control, medical and dental professionals can harness the therapeutic benefits of ozone while safeguarding patient well-being throughout the treatment process [10].

### 1.2. Applications of Ozone in Medicine

Several forms of utilizing medical ozone include topical, infiltrative, and systemic use. The topical use of ozone can be achieved using ozonated water or ozonated oils to affect the healing process or induce a germicidal effect. Infiltrative forms of ozone are used to treat several musculoskeletal diseases such as arthritis, myositis, fasciitis, tendonitis, neuritis, or myofascial pain. Such use of infiltrative ozone has increased over the years. This technique is quite promising as the infiltration uses a highly oxidizing gas with a good tissue diffusion capacity. For instance, intramuscular ozone applications at the paravertebral level were reported to treat chronic lower back pain, epicondylitis, acute and chronic polyarthritis (interphalangeal, sacroiliac joint, hips, and knees), carpal tunnel syndrome, and myofascial pain [11]. Systemic administration of ozone can be achieved through the indirect intravenous route, also known as autohemotherapy and rectal insufflation. Autohemotherapy, which has been used since 1950, includes treating up to 200 mL of previously isolated human blood with a gaseous combination of oxygen and ozone [12]. Ozone has been used to treat various unrelated illnesses, including ulcers, cutaneous infections, acute and chronic viral diseases, neoplasia, and vascular problems such as obstructive arteriopathies, venous insufficiency, and vascular degenerative diseases [13,14,15]. In 2022, Galluccio proposed that a therapy known as H–O–U, combining heating, ozonation, and UV light exposure, might have a therapeutic impact on managing Raynaud’s syndrome. This therapy reduced or stopped Raynaud’s episodes for at least three months [16]. Another way of administration is rectal insufflation, during which ozone and oxygen are given intravenously, followed by the absorption of an oxygen and ozone combination in the colon [17,18]. Interestingly, ozonated saline is an efficient irrigation solution for minimizing abscess development compared to regular saline solution and saline–cephalothin irrigation in treating fecal peritonitis in rats [19]. It was noted that rectal insufflation, as opposed to intravenous and intramuscular methods, was the least unpleasant, safest, and most practical way for administering ozone to 72 non-diabetic individuals with vascular occlusion due to atherosclerosis [20].

Ozone bagging entails surrounding the area to be treated with an airtight bag. The bag is pumped with ozone and oxygen, which the skin absorbs [21]. In 1967, Germany was the first country to employ this ion ozone treatment. It has been used to treat open wounds and ulcers like varicose, diabetic, and pressure sores since it was discovered to have a bactericidal effect, especially on staphylococcal, streptococcal, and protean infections. The purpose of an ion ozone generator is to produce steam that is then passed over a mercury vapor arc to ionize it, resulting in a combination of ionized water, ozone, and oxygen. A bactericidal effect was also seen when the technique was used in conjunction with antibiotics. The physiological effects observed can be considered to produce a sedative effect on sensory nerve endings and/or stimulation of the superficial blood flow [22].

### 1.3. Applications of Ozone in Dentistry

In dentistry, the ozone has a severely disruptive effect on cariogenic bacteria, eliminating acidogenic bacteria [23,24,25,26]. Pyruvic acid is the most potent acid created by acidogenic bacteria during cariogenesis. Ozone can decarboxylate this acid to produce acetic acid, which is “much less acidic than pyruvic acid”, raising the pH and causing carious lesions to mineralize under more alkaline conditions [27]. Moreover, ozone effectively primes exposed dentinal tubules to promote mineral penetration and subsequent sealing, rapidly decreasing dentin sensitivity. Ozone also eliminates bacterial contamination in the exposed dentinal tubules [27]. The aim of this review is to provide a summary of the current uses of ozone in medicine and dentistry.

## 2. Methods

PubMed, Cochrane, and Google Scholar search engines were used to conduct an electronic search of scientific papers published between 2012 and 2023. The search terms used were ozone, clinical applications, medicine, and dentistry.

## 3. Results

**Figure 1:**
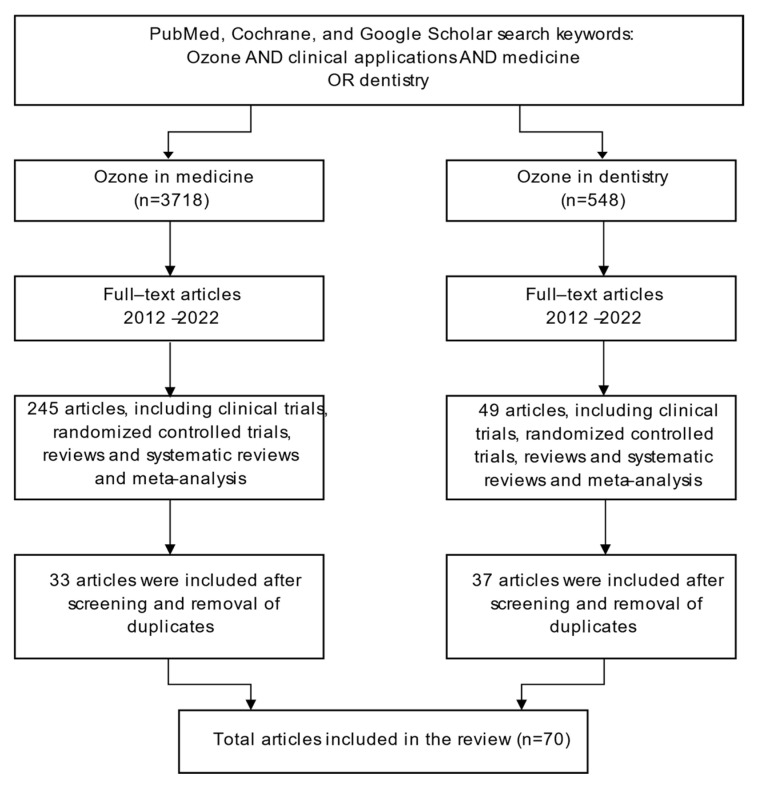
Flowchart of the search for articles included in the current review. n = number of articles.

A total of 3718 papers were identified in medicine and 548 papers in dentistry. Full-text articles between 2012 and 2023 were selected, where a total of 245 and 49 articles including clinical trials, randomized controlled trials, reviews, systematic reviews, and meta-analysis were reviewed in medicine and dentistry, respectively. After screening and removal of duplicates, a total of 70 articles (33 and 37 articles in medicine and dentistry, respectively) that described the use of ozone therapy in medicine and dentistry were included in the present review (Figure 1).

## 4. The Uses of Ozone in Medicine

German troops’ gaseous post-traumatic gangrene, infected wounds, mustard gas burns, and fistulas were all treated with ozone gas during World War I. From 1880 to 1932, ozone therapy was acknowledged as an alternative medicine in the USA. By the end of the 20th century, in 16 nations, ozone therapy had been accepted as a form of treatment [28]. Werner von Siemens created the first ozone generator in Germany as early as 1857, and C. Lender published its medicinal application for blood purification in 1870 [2]. Its application in treating ischemic disorders, orthopedic diseases, bacterial, viral, and fungal infections, as well as dermatological, pulmonary, renal, hematological, and neuro-degenerative diseases, has been studied. It can react with blood components and positively affect oxygen metabolism, cell energy, immunomodulator system, antioxidant defense system, and microcirculation [5]. It is interesting to note that people with inflammatory bowel diseases, such as ulcerative colitis and Crohn’s disease, as well as persistent bacterial diarrhea, have been treated with ozone. Ozone treatment is now a recognized therapeutic option in several nations, but to determine its efficacy, double-blind clinical studies are needed [2].

As mentioned, ozone gas can be administered systemically using a variety of techniques, including autohemotherapy, rectal insufflations “enema”, oxygen “bagging”, and ozonated olive or sunflower oils [29]. The potential for air emboli is one of the side effects of ozone if directly injected intravenously and can be fatal. They have few antioxidant and neutralizing resources; thus, respiratory mucosa, blood, and eyes are particularly vulnerable to ozone. A lipid ozonation product is created when the ozone is combined with the lipids in the fluid layer that coats the lungs, relaying ozone’s damaging effects to deeper tissue strata that ozone alone is unable to reach. Ozone should never be ingested, and a hospital emergency room must have a suitable monitor, ozone destroyer, and air purifier [29]. Acute alcohol intoxication, a recent myocardial infarction, hemorrhage from any organ, pregnancy, hyperthyroidism, thrombocytopenia, severe anemia, and ozone allergy are all contraindications to using ozone therapy [5].

Ozone has been reported to treat more than 100 different diseases due to its great health benefits in the medical field [30,31]. Its antioxidant activity was found to aid in reducing blood cholesterol levels and complementary treatment of hypoxic and ischemia-associated diseases [32,33,34,35,36]. However, the absence of standardization in ozone treatment protocols makes it challenging to compare the outcomes, making it difficult to draw reliable conclusions or suggestions.

### 4.1. Effect of Ozone on Pain Management

Ozone therapy showed promising results in pain medicine. This was due to injected ozone’s anti-inflammatory, analgesic, and anti-edema properties. One of the possible mechanisms of pain inhibition is through the oxidation of the algogenic receptors and activation of the anti-nociceptive system [37]. Additionally, a single peripheral injection of medical ozone decreased mechanical allodynia triggered by sciatic damage and activation of pro-inflammatory caspases in mice [38,39]. Additionally, other inflammatory knee pathologies were explored, where peritendinous ozone injections showed success in patients with refractory knee tendinopathies and athletes with “jumper’s knee” who did not recover after receiving standard treatments [40,41]. This is similar to the reported data of Manzi and Raimondi, where injected ozone showed high and rapid resolution of pain in patients suffering from patellofemoral chondromalacia [42]. In addition, ozone therapy showed some muscle relaxant effects and can be used to treat painful muscle hypertonia [43].

### 4.2. Effect of Ozone on Musculoskeletal Diseases

Typically, ozone therapy is used in conjunction with other local or systemic therapies [7]. Combinational therapy using ozone applications showed synergistic effects in several musculoskeletal diseases. For example, ozone therapy combined with shock waves showed great success in treating calcifying shoulder tendinitis [44]. Furthermore, injected ozone exhibited higher efficacy in the treatment of subacromial tendinopathy when compared to steroid injections or physiotherapy [45,46]. Likewise, ozone therapy showed more favorable outcomes when used in patients with neck pain, upper limb paresthesias, peripheral vertigo, and headaches compared to mesotherapy [47]. Patients suffering from lumbar sciatic pain due to disk protrusions showed better improvement in their functional capacity when treated with intradiscal and intra-foraminal ozone injections as compared to steroids [48,49,50,51,52]. Such favorable outcomes, the safety of the technique (whether paravertebral or intradiscal), and surgery complications have made more physicians consider ozone therapy, especially in the case of failure of standard treatment.

Ozone therapy was also utilized in the treatment of rheumatoid arthritis. Intra-articular ozone injections inhibited synovitis and decreased the expression of inflammatory cytokines such as TNF–α in animal models. Thus, ozone may be used to treat rheumatoid arthritis patients, especially those suffering from excessive inflammation and joint infiltrations [53,54]. In addition, systemic ozone therapy in combination with methotrexate for rheumatoid arthritis treatment was reported to enhance its efficacy and decrease the side effects [55]. In addition, systemic ozone therapy was identified to reduce IL–1β levels, while intra-articular administration decreased IL–8 levels in rheumatoid arthritis patients [54,56]. Likewise, ozone was proposed to relieve the pain of affected knees in osteoarthritis patients [30,31].

### 4.3. Effect of Ozone on Infectious Disease Management

Ozone was also reported to be effective in the treatment of infectious diseases. It was identified to disinfect extracorporeal blood samples of human immune-deficiency virus (HIV) in vitro by reducing the HIV p24 core protein. However, ozone therapy was ineffective when applied to acquired immunodeficiency syndrome (AIDS) patients [57]. Moreover, ozone therapy was proposed to treat severe acute respiratory syndrome (SARS) in combination with the standard therapeutic protocols [26]. This was attributed to the peroxidation damage induced by ozone to the viral capsid and the reproductive cycle. Regarding bacterial infections, ozone-induced oxidation of phospholipids and lipoproteins disrupted the integrity of the bacterial cell envelope [30,31]. Table 1 shows recent studies featuring the use of ozone in medicine.

## 5. The Uses of Ozone in Dentistry

Dr. Fisch (1899–1966) was the first dentist to employ ozonated water in his practice. Dr. Payr, a German surgeon, received it from him and began using it in surgery after that. He announced his findings at the 59th Congress of the German Surgical Society in Berlin (1935) [21].

### 5.1. Effect of Ozone on Soft Tissues

In dental surgery, ozone can be used as a gas or dissolved in water to limit bacterial growth, improve homeostasis, and increase local oxygen supply by increasing blood flow [64]. Similarly, ozone gas has been utilized to treat illnesses, including herpes and aphthous ulcers. It hastens the healing process and shortens the illness’s clinical course. Herpes vanished after three days if ozone gas was given early and frequently, while aphthous ulcers vanished after one day [65]. Additionally, the use of ozone is well indicated in all stages of gingival and periodontal diseases due to its beneficial biological effects, antimicrobial activity, oxidation of biomolecule precursors and microbial toxins implicated in periodontal diseases, and its healing and tissue regeneration properties [66].

### 5.2. Effect of Ozone on Dental Hard Tissues

Ozone has the potential to lower the bacterial population in active carious lesions. To prevent or delay the need for tooth repair, it may temporarily stop the progression of cavities in enamel or dentin [67]. Some of the studies that are now accessible evaluated how ozone affected open caries, non-cavitated occlusal carious lesions, pit and fissure caries, and primary root caries. With short-term follow-up, the results demonstrated a considerable decrease in the number of microorganisms in the carious lesions in vivo and in vitro [68,69,70,71,72,73].

As a result of the oxidative action of ozone against bacterial strains such as *Enterococcus faecalis* [74] and *Candida albicans* [75], ozone is indicated for use in endodontic therapy [76]. Another use of ozone gas is reducing dentin hypersensitivity [77].

Ozone is used in implantology to aid bone regrowth. The socket is traditionally prepared, and over the next 40 s, ozone is bubbled into the prepared socket. An implant is then inserted into the socket. This lessens the risk of infection and promotes bone regrowth [78]. Studies have also revealed encouraging reports of regeneration and the eradication of infection around the implant in cases of peri-implantitis [78,79].

### 5.3. Effect of Ozone on Physical Properties of Enamel and Dentin

In the same way, Marchesi et al., 2012, assessed whether gaseous ozone application affected the microleakage of two dental sealants or the immediate enamel bond strength [80]. The results showed that ozone did not significantly weaken the enamel’s bond strength or cause microleakage to increase. They concluded that ozone gas did not affect the tested materials’ ability to adhere; therefore, one can clean the enamel surface before applying a dental sealant without compromising its ability to perform clinically.

According to Pires et al., 2013, in evaluations of the effect of ozone pretreatment on the shear strength of an etch-and-rinse and a self-etch system to enamel, as well as an analysis of the corresponding failure modes, neither adhesive’s shear bond strength values tested on enamel were affected by the prior application of ozone gas [81].

Floare et al., 2022, studied the impact of ozone (O_3_) treatment on the microstructural changes in tooth enamel after the treatment at different time intervals [82]. The results showed that exposure to O_3_ for 40–50 s enhanced enamel microhardness and ensured a rate of remineralization of between 96.82 and 97.38%. They concluded that using O_3_ as an alternative therapy to classical solutions may be a viable solution in dentistry.

### 5.4. Ozone in the Management of Non-Cavitated Pit and Fissure Caries

Several studies evaluated the effect of different application times of ozone gas that ranged from 10 to 40 s on primary pit and fissure caries. The remineralization and progression or regression of caries were measured directly after ozone application or after a follow-up period varying from 2 to 12 months. The change in the mean DIAGNOdent reading and the combination of the clinical severity scores and DIAGNOdent readings were evaluated [69,70,83]. In other studies, an electric caries meter was used to measure the carious lesion’s remineralization [84,85]. These studies showed a decrease in the DIGNOdent reading and clinical severity scores and an increase in the electrical caries meter measurement, indicating caries regression.

To treat non-cavitated occlusal fissure carious lesions in first permanent molars, El Meligy and Almushayt examined the efficacy of ozone gas and fissure sealant [83]. The trial enrolled fifty patients. Ozone was found to be equally beneficial as the fissure sealant 12 months after treating first permanent molars with active, non-cavitated occlusal fissure carious lesions.

In a split-mouth study, Johansson et al. assessed the impact of ozone and fluoride varnish on occlusal caries in primary molars [84]. Treatment with ozone or fluoride varnish did not halt caries development in cavitated lesions. Children with low and moderate caries risk had non-cavitated lesions that either showed minimal or no progression after both treatments. The use of ozone or fluoride varnish treatments in this regimen must be questioned to stop the advancement of caries in primary molars. These treatments are used in addition to the regular use of fluoridated toothpaste.

Unal and Oztas studied the activation of remineralization following the administration of three fissure sealants (FSs) on non-cavitated early caries, either alone or in combination with gaseous ozone (GO), and assessed the clinical success of FSs [71]. They concluded that GO, along with Aegis FS, demonstrated the highest levels of remineralization and that at the end of 12 months, its clinical success was higher than that of other FSs.

### 5.5. Ozone in the Management of Cavitated Occlusal Carious Lesions

Using stepwise excavation, Safwat et al. assessed the clinical changes in the dentin of deep carious lesions in young permanent molars after ozone exposure with and without using a remineralizing solution [86]. Their findings showed that the dentin color and consistency of young permanent molars were unaffected by ozone application through stepwise excavation. Monitoring caries activity using DIAGNOdent was unreliable.

Using a tooth cavity model, Sancakli et al. assessed the efficacy of antibacterial surface pretreatment techniques against *Streptococcus mutans* (*S. mutans*) within the diseased dentin surface [73]. They concluded that antibacterial effects against S. mutans were produced by the use of the Er:YAG and KTP lasers, as well as their additional combinations, during the cavity pretreatment procedure with chlorhexidine and ozone treatments, whereas the use of chlorhexidine and antibacterial dentin bonding only produced the highest antibacterial effects.

The antibacterial efficiency of ozone therapy on cariogenic bacteria was assessed by Düzyol [87]. Ozone and chlorhexidine digluconate (CHX) treatment groups were created from 40 children with deep caries in the permanent first molar. Samples of cariogenic dentin were taken from permanent molars before and after 120 s treatment with ozone and 60 s treatment with 2% CHX solution. The colonies of *S. mutans* and *Lactobacillus* sp. were counted after 48 h of phosphate buffer incubation. Both zone and CHX had antimicrobial effects against *S. mutans* and *Lactobacillus* sp.; however, they had significant differences (*p* < 0.05). The amount of growth in the *Lactobacillus* sp. group was substantially reduced in the CHX group compared to the ozone group (*p* < 0.05). They concluded that cariogenic bacteria could be cleaned using ozone therapy. Krunić et al., on the other hand, concluded that the antibacterial effect of ozone on residual bacteria after inadequate caries eradication was comparable to that of 2% chlorhexidine [88]. Additionally, when treating deep carious lesions with an incomplete caries removal technique, ozone shows promise as a biocompatible and efficient cavity disinfectant.

Almaz and Sönmez reviewed clinical and in vitro research to determine the efficacy of ozone therapy in the treatment and prevention of caries [70]. Ozone has been cited as a promising alternative to traditional caries control techniques in most clinical research. Ozone has been proven in a few studies to be ineffective at stopping caries and minimizing bacteria in open occlusal carious lesions. Ozone may be a valuable technique to lessen and control oral infectious germs in dental plaque and dental cavities. The outcomes of in vitro investigations, however, are debatable. While some researchers claimed that ozone therapy had little to no impact on the survival of bacteria, others claimed it was quite efficient at eradicating Gram-positive and Gram-negative oral germs. Therefore, more research is necessary before ozone is approved as a substitute for current approaches to managing and preventing caries.

The Cochrane library sponsored a systematic review of ozone in clinical dentistry in 2004 to evaluate the efficiency of ozone gas in delaying or halting the spread of dental caries [23]. The authors concluded that there is insufficient solid proof that ozone administration arrests or reverses the decay process. The use of ozone gas in a primary care setting for the treatment of dental caries also requires additional suitable quality evidence. A similar systematic and meta-analysis review was carried out by Santos et al. to determine the efficacy and safety of ozone therapy for the treatment of dental caries [89]. They came to the same conclusion as the Cochrane library.

### 5.6. Role of Ozone in Oral Medicine and Periodontology

Ozone therapy’s potential efficacy against periodontal pathogens and Candida species is indeed an essential aspect to consider in the context of its application in dentistry. Numerous studies have investigated the antimicrobial properties of ozone and its potential as an adjunctive treatment in various oral conditions, including periodontal diseases and oral candidiasis [90,91]. When ozone is applied to the oral cavity, it can exert antimicrobial effects by disrupting the cell walls of microorganisms and interfering with their metabolic processes. This makes ozone an attractive option for treating infections caused by periodontal pathogens and Candida species, which are known to play significant roles in periodontal disease and oral thrush, respectively [92,93,94,95]. Several research papers have reported positive outcomes when using ozone therapy in conjunction with conventional dental treatments. These studies have shown that ozone can effectively reduce bacterial and fungal loads, leading to improved clinical outcomes in patients with periodontal infections and oral candidiasis [75,96,97,98].

### 5.7. Ozone and Dental Unit Water Lines

Contamination of the dental unit water line (DUWL) has become an issue [99,100]. While the unit is not in use, the water becomes stagnant. Dental operations may expose healthcare workers to bacteria, spatter, and aerosols [100]. Molds, bacteria, and yeasts that are dangerous to the healthcare provider and other patients during therapy have been found by Szymanska in biofilms [101]. From the mains water, opportunistic pathogens were cultivated. Another study claimed that DUWL biocides could negatively impact the resin’s ability to adhere to the enamel [102]. Ozone has been used to clean water because of its effectiveness and absence of adverse side effects. According to results published by Kohno et al., acidic electrolyzed water could be used as a suitable defense against bacterial contamination of the DUWL [103]. Ozone was applied briefly and at a very low dose, but it nevertheless reduced biofilms and viable bacteria in model DUWLs by 57% and 65%, respectively [104]. Table 2 shows recent studies featuring the use of ozone in dentistry.

## 6. Safety, Toxicity, and Contraindications of Ozone

Ozone is a valuable medicinal agent, comparable to many other drugs, but it only works when used as prescribed and under controlled conditions. The only tool utilized to treat dental caries in the literature that has been published is HealOzone. Other ozone production techniques have been utilized in controlled in vitro conditions. However, they come with significant risks to the operator and patient safety. HealOzone is the only approved device for intraoral applications because it is designed with a delivery system that ensures a complete and proper ozone seal. In addition, none of the published clinical studies mentioned any adverse outcomes [89].

Millar and Hodson assessed the two ozone-generating devices’ safety for dental use [113]. These were HealOzone and the Ozi-cure devices. The two devices were compared based on how much ozone leaked after applying gas. When used without enough suction, the researchers discovered that the Ozi-cure device allowed ozone to accumulate in the pharynx at high quantities (1.33 ± 0.52 ppm) over the authorized levels (0.01 ppm). With the HealOzone device, there were no records at all. Therefore, they concluded that the HealOzone was safe, but the Ozi-cure device should not be used.

Adverse effects of ozone reactivity are due to the peroxidation or generation of free radicals and the production of lipid ozonation products [114,115]. This triggers the activation of lipases and the release of inflammatory mediators [115]. Most current therapies using ozone are quite safe when used with great precision. However, minor adverse effects include nausea, headaches, fatigue, epiphora and upper respiratory irritation, rhinitis, a cough, and vomiting [116,117]. Also, it was reported that a vagal reaction is an adverse effect that is associated with pain during ozone infiltration. Therefore, slow ozone administration is recommended to prevent such side effects, especially at high concentrations [118].

It is worth mentioning that there are few contraindications for using ozone as a therapeutic modality. Patients suffering from deficiency of the glucose–6 dehydrogenase phosphate (G6PD) enzyme (favism) are contraindicated for ozone therapy. This is because this enzyme is critical for the oxidation of the lipoperoxides and the function of the glutathione system [119]. Other contraindications include uncontrolled hyperthyroidism, thrombocytopenia, cardiovascular problems, seizures, severe anemia, severe myasthenia, ozone allergy, recent myocardial infarction, hemorrhage from any organ, and acute alcohol intoxication [120]. Moreover, ozone therapy is not recommended in pregnancy, as it has not been thoroughly tested [121].

Johansson et al. investigated the sealing capacity of the HealOzone device and ozone leakage during accidental displacement of the cup [122]. The ozone gas concentrations near the delivery cups were measured before and after 10–20 s application cycles and during and after cup displacements during the cycle. The results demonstrated that the ozone application cycles with relocated cups have significantly higher leakage levels than continuous complete cycles compared to the room’s background ozone concentrations. They concluded that a similar shift near the delivered cups coincided with the change in background ozone levels within the room. The overall measured ozone leakage values were minimal after delivery cycles that were regularly operating. Thus, the delivery method can be regarded as secure.

Ozone should never be inhaled due to the overwhelming research demonstrating how vulnerable the bronchial–pulmonary system is to it. Mucosal cells in the respiratory tract lining fluid are highly susceptible to oxidation because the fluid is made up of a very thin, watery layer with very little antioxidant content. As a result, there was a pulmonary embolism during the direct intravenous delivery of O_2_/O_3_, which the European Society of Ozone Therapy has prohibited since 1983. In ozone intoxication, patients must lie down, inhale humid oxygen, and take ascorbic acid/vitamin C, vitamin E, and N–acetylcysteine [120].

## 7. Conclusions

Ozone therapy has shown promising potential as an alternative or complementary treatment option for various medical conditions. However, clinical research has not entirely lived up to this potential in dentistry. Therefore, well-designed clinical trials with sufficient sample sizes, lengthy follow-up periods, and standardized assessment methods are required to examine the potential use of ozone as a therapeutic option in dentistry.

Standardization of ozone therapy protocols is essential to establish uniformity in treatment procedures, dosages, and administration routes. This will not only enhance the safety and efficacy of the therapy but also allow for more robust comparisons between different studies. Additionally, standardized documentation practices will enable researchers and clinicians to better evaluate and synthesize the available evidence, ultimately contributing to evidence-based decision making.

## Figures and Tables

**Table 1:** Recent studies featuring the use of ozone in medicine.

Authors and Publication Date	Study Title	Journal	Sample Size	Conclusion	Reference
Fitzpatrick et al., 2018	Ozone therapy for the treatment of chronic wounds: a systematic review	*Int. Wound. J.*	Nine studies (n = 453 patients)	Compared with standard care, ozone therapy as an advanced wound care treatment may improve the proportion of chronic wounds healed in a shorter amount of time, but further research is required	[58]
Andrade et al., 2019	Effectiveness of ozone therapy compared to other therapies for low back pain: a systematic review with meta-analysis of randomized clinical trials	*Braz. J. Anesthesiol*.	Six clinical trials	Ozone therapy used for six months for lumbar pain relief is more effective than other therapies	[59]
Sconza et al., 2020	Oxygen–ozone therapy for the treatment of knee osteoarthritis: a systematic review of randomized controlled trials	*Arthroscopy*	11 studies involving 858 patients in total (629 female and 229 male) were included	On the basis of the data available, oxygen–ozone therapy has, however, proven to be a safe approach with encouraging effects in pain control and functional recovery in the short–middle term	[60]
Sconza et al., 2021	Oxygen–ozone therapy for the treatment of low back pain: a systematic review of randomized controlled trials	*Eur. Rev. Med. Pharmacol. Sci.*	15 studies involving 2597 patients in total were included	Oxygen–ozone therapy has proven to be a safe treatment with beneficial effects in pain control and functional recovery at short- to medium-term follow-up	[61]
Serra et al., 2023	The role of ozone treatment as integrative medicine. an evidence and gap map	*Front. Public. Health*.	26 systematic reviews were characterized	Ozone treatment contributes to controlling pain, infections, inflammation, and wound healing, as well as increasing the quality of life. No serious adverse effects were related	[62]
Setyo Budi et al., 2022	Ozone as an adjuvant therapy for COVID-19: a systematic review and meta-analysis	*Int. Immunopharmacol.*	13 studies were included in this review	The beneficial effect of ozone in COVID-19 management is limited to the improvements of laboratory parameters among severe patients. Additionally, no serious adverse event was reported following ozone therapy, suggesting its high safety profile	[63]

**Table 2:** Recent studies featuring the use of ozone in dentistry.

Authors and Publication Date	Study Title	Journal	Sample Size	Conclusion	Reference
Isler et al., 2018	Effects of laser photobiomodulation and ozone therapy on palatal epithelial wound healing and patient morbidity	*Photomed. Laser. Surg.*	36 patients	Significantly improves the healing of palatal lesions	[105]
Al-Omiri et al., 2018	Randomized clinical trial on the comparison of bleaching outcomes using either ozone or hydrogen peroxide	*Quintessence. Int.*	32 participants	Teeth containing similar bleaching outcomes	[106]
Durmus et al., 2019	Effectiveness of the ozone application in two-visit indirect pulp therapy of permanent molars with deep carious lesion: a randomized clinical trial	*Clin. Oral. Investig.*	105 lower first molar teeth	Significant effect in reducing microorganisms	[107]
Uraz et al., 2019	Ozone application as adjunctive therapy in chronic periodontitis: clinical, microbiological and biochemical aspects	*J. Dent. Sci.*	18 periodontitis patients	Ozone therapy had no further benefits in terms of clinical, microbiological, or biochemical markers	[108]
Matys et al., 2020	Effect of ozone and diode laser (635 nm) in reducing orthodontic pain in the maxillary arch: a randomized clinical controlled trial	*Lasers. Med. Sci.*	76 patients	Diode lasers had a significant pain-relieving effect while ozone did not relieve pain	[109]
Grocholewicz et al., 2020	Effect of nano-hydroxyapatite and ozone on approximal initial caries: a randomized clinical trial	*Sci. Rep.*	92 participants	A combination of nano-hydroxyapatite gel and ozone therapy produced the best effect in remineralizing enamel	[110]
Al-Omiri et al., 2021	Treatment of symptomatic, deep, almost cariously exposed lesions using ozone	*Sci. Rep.*	84 participants	Ozone applied to partially excised cavities prior to repair alleviates pain	[111]
M. Serag Eldien and Fathy Hassabou, 2022	Clinical and cytological assessment of platelet-rich fibrin versus topical ozonated oil in palatal wound healing after free gingival graft harvesting: randomized controlled trial	*J. Oral. Maxillofac. Surg. Med. Patho.*	39 patients	It has a significant improvement in wound healing re-epithelialization	[112]

## Data Availability

The data presented are available in the article.
